# Opisthorchiasis with proinflammatory cytokines (*IL-1β* and *TNF-α*) polymorphisms influence risk of intrahepatic cholangiocarcinoma in Thailand: a nested case-control study

**DOI:** 10.1186/s12885-018-4751-5

**Published:** 2018-08-23

**Authors:** Supannee Promthet, Nopparat Songserm, Somkiattiyos Woradet, Chamsai Pientong, Tipaya Ekalaksananan, Surapon Wiangnon, Akhtar Ali

**Affiliations:** 1 Department of Epidemiology and Biostatistics, Faculty of Public Health, Khon Kaen University, Khon Kaen, Thailand; 2 ASEAN Cancer Epidemiology and Prevention Research Group, Khon Kaen University, Khon Kaen, Thailand; 3 Department of Community Health, Faculty of Public Health, Ubon Ratchathani Rajabhat University, Ubon Ratchathani, Thailand; 4 Department of Public Health, Faculty of Health and Sports Science, Thaksin University, Phatthalung, Thailand; 5 Department of Microbiology, Faculty of Medicine, Khon Kaen University, Khon Kaen, Thailand; 6 HPV & EBV and Carcinogenesis Research Group, Khon Kaen University, Khon Kaen, Thailand; 7 Department of Biological Science, The University of Tulsa, Tulsa, OK USA

**Keywords:** *IL-1β*, *TNF-α*, Polymorphism, Opisthorchiasis, Cholangiocarcinoma

## Abstract

**Background:**

Chronic inflammation and repeated infection with *Opisthorchis viverrini* (*O. viverrini*) induces intrahepatic cholangiocarcinoma (ICC). Inflammatory cytokines such as interleukin (IL) and tumor necrosis factor (TNF) are substances in the immune system that promote inflammation and causes disease to progress. Genes that help express proinflammatory cytokines can affect an individual’s susceptibility to disease, especially in cancer-related chronic inflammation. This study aimed to investigate risk factors for ICC with a focus on opisthorchiasis and polymorphisms of proinflammatory cytokines (*IL-1β* and *TNF-α*).

**Methods:**

This study was a nested case-control study within a cohort study. 219 subjects who developed a primary ICC were identified and matched with two non-cancer controls from the same cohort based on sex and age at recruitment (±3 years). An *O. viverrini*-IgG antibody was assessed using enzyme linked immunosorbent assay. *IL-1β* and *TNF-α* polymorphisms were analyzed using a polymerase chain reaction with high resolution melting analysis. Associations between variables and ICC were assessed using conditional logistic regression.

**Results:**

Subjects with a high infection intensity had higher risk of ICC than those who had a low level (OR = 2.1; 95% CI: 1.2–3.9). Subjects with all genotypes of *TNF-α* (GG, GA, AA) and high infection intensity were significantly related to an increased risk of ICC (*p* < 0.05).

**Conclusions:**

Polymorphisms of *IL-1β* and *TNF-α* are not a risk of ICC, but an individual with *O. viverrini* infection has an effect on all genotypes of the *TNF-α* gene that might promote ICC. Primary prevention of ICC in high-risk areas is based on efforts to reduce *O. viverrini* infection.

## Background

The incidence rate of intrahepatic cholangiocarcinoma (ICC) in the Northeast – the highest incidence in Thailand – from the last report, *Cancer in Thailand, Vol. VII, 2007–2009*, showed that it is the most common cancer in men (age-standardized incidence rate, ASR = 67.1 per 100,000) and the third most common cancer in women (ASR = 30.9). In Khon Kaen, located in Northeast Thailand, the incidence of ICC is ASR 57.4 in men, and 23.1 in women per 100,000 [1].

ICC is a multi-factor and inflammation-linked disease. Chronic inflammation and repeated infection with liver fluke, *Opisthorchis viverrini* (*O. viverrini*), induces ICC development [2–4]. Inflammatory cytokines increase inflammation and can cause diseases to progress rapidly. Interleukin-1β (IL-1β) and tumor necrosis factor-β (TNF-α) are inflammatory mediators that have been implicated in carcinogenesis due to its participation in chronic inflammatory diseases [5] including ICC [6]. The genesis of ICC is a multistep process, which required interaction between mutated biliary epithelial cells and environmental factors [7]. During ICC development, chronic inflammation of the bile duct caused by *O. viverrini* infection can induce the epithelial cells to produce a variety of cytokines, including IL-6, IL-8, TGF-β and TNF-α [8]. This can cause bile duct epithelial cell proliferation and impaired epithelial barrier function. As a result, somatic mutations occur in several tumor-related genes, leading to cholangiocarcinogenesis [6, 8–10]. Some studies reported the expression of TNF-α associated with ICC [6, 9], but genetic polymorphisms in *TNF-α* and ICC risk has not been explored so far and requires further study. IL-1β is a proinflammatory cytokine with multiple biological effects [11]. It has been implicated as an important factor for cancer progression. Genetic polymorphisms in *IL-1β* are associated with gastric cancer [12], hepatitis C virus [13–15] and hepatitis B virus [16] linked with hepatocellular carcinoma (HCC). However, there is no study of *IL-1β* genetic polymorphisms on ICC risk. The present study therefore aims to investigate risk factors for ICC and inflammation-linked cancer, and focus on opisthorchiasis and polymorphisms in proinflammatory cytokines (*IL-1β* and *TNF-α*) to assess whether these genes are involved in opisthorchiasis-related ICC risk.

## Methods

### Study design

This study was a part of a larger study known as the Khon Kaen Cohort Study (KKCS) that was previously conducted [17–20]. This was a suitable platform to test the hypothesis on a host-environment interaction influence risk of ICC. Briefly, positive subjects of ICC (ICD-10: 22.1) and a sample of non-affected controls that were enrolled participants in the KKCS were used in these experiments.

### Study subjects

Among the 23,584 subjects admitted in the KKCS (male 32.6%: female 67.4%), 219 cohort participants (0.9%) that developed a primary malignancy of the intrahepatic bile ducts of the liver (C22.1) were identified (male 57.0%: female 43.0%). Since ICC is rarely diagnosed by liver biopsy and histopathology (6.9%), the criteria for inclusion as a case included diagnosis at least by ultrasound, with or without contrast radiology (9.6%) and tumor markers such as CA19–9 (83.5%). The vital status and date of death for potential cases were ascertained by linkage to the file of deaths in Thailand, in the database of the National Health Security Office (NHSO), together with the demographic database of Ministry of Interior. All ICC cases died within 2 years of diagnosis. Two non-cancer controls from the same cohort population were randomly selected for matching with each case based on sex and age at recruitment (±3 years).

### Detection of *O. viverrini*-IgG antibody

Detection of *O. viverrini*-IgG antibody was assessed at the parasitological laboratory, Faculty of Medicine, Khon Kaen University, Thailand. The indirect enzyme linked with immunosorbent assay (ELISA) was used to analyze a serum of cases and their matched controls as reported before [21, 22]. The samples were analyzed as duplicates with the optical density (OD) at 620 nm under the ELISA reader. The mean OD was used as the cut-off value, OD ≤0.24 and OD > 0.24.

### Analysis of *IL-1β* and *TNF-α* polymorphisms

Genomic DNA was extracted from buffy coat fractions of 170 cases (77.6%) of 219 eligible ICC cases and 355 (81.0%) of 438 matched controls using the standard protocols of Genomic DNA mini Kit with Proteinase K (Geneaid Biotech).

**Fig. 1:**
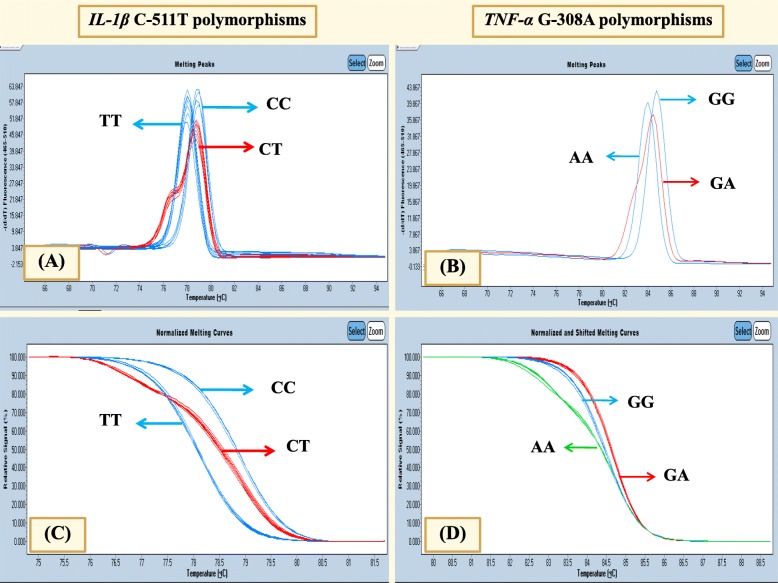
Polymorphisms in *IL-1β* C-511T and *TNF-α* G-308A were analyzed using the polymerase chain reaction with high resolution melting analysis (PCR-HRM)

The amplification of *IL-1β* C-511 T was achieved by using two primers, [F]:5′- AATTTCTCAGCCTCCTACTTC-3′ and [R]: 5′- GTTTGGTATCTGICCGTTTC-3′. The *TNF-α* G308A gene was amplified by using [F]: 5′- TAGGTTTTGAGGGGCATG -3′ and [R]: 5′- CTGGGICCCTGACTGATTT-3′ in a PCR reaction. Both genetic polymorphisms were performed in a LightCycler® 480 Real-Time PCR System with a final volume of 20 μl containing 10 μl of master mix, 4.4 μl of H_2_O, 3 mM of MgCl_2_, 0.3 μM of each primer and 200 ng of the DNA template. The data of high resolution melting analysis was analyzed using the LightCycler 480® Gene Scanning Software version 1.5 (Roche). Normalized melting curves and melting peaks of sequence variation were evaluated and compared with the wild-type sample. Different plots of melting peaks are illustrated in Fig. 1a for *IL-1β* C-511 T and Fig. 1b for *TNF-α* G-308A. Sequence variations were distinguished by the different shape of melting curves (Fig. 1c for *IL-1β* C-511T and Fig. 1d for *TNF-α* G-308A). To improve the genotyping quality and validation, 10% of random genotyping samples were confirmed by the PCR with restriction fragment length polymorphism techniques (PCR-RFLP).

### Statistical analysis

The link between *O. viverrini* infection intensity and proinflammatory cytokines polymorphisms (*IL-1β* and *TNF-α*) with the risk of developing ICC and odds ratios (ORs) were assessed and estimated at the 95% confidence and conditional logistic regression. The information about smoking, alcohol, and diet that was used for the adjusted ORs from the multivariate analysis in Table 2 has been previously reported [19, 20]. Possible modifications of the effects of *O. viverrini* infection intensity by proinflammatory cytokines polymorphisms (*IL-1β* and *TNF-α*) were also analyzed. Results that showed a *p*-value < 0.05 were statistically significant from the native control.

## Results

Out of 23,584 participants, 219 (0.9%) subjects developed ICC. This was comprised of 92 females and 127 males and with a median age of 57 years (Table 1). Additionally, there were two controls of the same sex and age for each case.

**Table 1:** General characteristics of intrahepatic cholangiocarcinoma cases and their matched controls

Variables	Cases		Controls	
	*n* = 219	%	*n* = 438	%
Sex				
Female	92	42.0	184	42.0
Male	127	58.0	254	58.0
Age at recruitment (years)				
30–39	7	3.2	13	3.0
40–49	34	15.5	70	16.0
50–59	99	45.2	199	45.4
60–69	79	36.1	156	35.6
Median (min: max)	57 (31: 69)		57 (30: 70)	
Education level				
Illiterate	26	11.9	15	3.4
Primary school	185	84.5	398	90.9
Secondary school or higher	8	3.7	25	5.7
Marital status				
Single	4	1.8	11	2.5
Married	173	79.0	362	82.7
Divorced	14	6.4	20	4.6
Widowed	28	12.8	45	10.3
Occupation				
Farmer	195	89.0	399	91.1
Government official	5	2.3	16	3.7
Commercial	15	6.9	16	3.7
General employee	4	1.8	7	1.6
Household income per year (Baht)				
Less than 60,000 (Low)	213	97.3	418	95.4
60,001–119,999 (Medium)	4	1.8	10	2.3
≥ 120,000 (High)	2	0.9	10	2.3
Median (min: max)	10,000 (1200: 120,000)		10,000 (1000: 360,000)	
*O. viverrini* egg in stool				
Negative	115	62.2	230	62.2
Positive	70	37.8	140	37.8

Table 2 shows the adjusted odds ratios (OR) and 95% CIs from the multivariate analyses, including the 10 factors identified as increasing risk in univariate analysis of ICC associated with *O. viverrini* infection intensity, *IL-1β* and *TNF-α* polymorphisms. Participants who had the *O. viverrini*-IgG antibody (OD > 0.24) possessed a higher risk for ICC than those who did not (OD ≤0.24) (adjusted OR 2.1; 95% CI: 1.2–3.9). In comparison, participants with TT variant of *IL-1β* C-511 T polymorphisms had a decreased risk of ICC (adjusted OR 0.4; 95% CI: 0.2–0.8). Results of interactions between *O. viverrini* infection intensity and *IL-1β* and *TNF-α* polymorphisms on the risk of ICC have been shown in Table 3. Participants with *TNF-α* in all genotypes (GG, GA, AA) who had high infection intensity (IgG antibody > 0.24) had an increased risk of ICC and were statistically significant (OR 2.1 for GG wild-type, OR 2.4 for GA heterozygote and OR 2.8 for AA variant). There were no interactions between *O. viverrini* infection intensity and the polymorphisms of *IL-1β* and *TNF-α* that influenced the risk of ICC.

**Table 2:** Odds ratios for intrahepatic cholangiocarcinoma associated with *O. viverrini* infection intensity, and with *IL-1β * and *TNF-α* polymorphisms

Variables	Cases		Controls		OR^a^	95% CI	*P*-value	OR^b^	95% CI	*P*-value
	n	%	*n*	%						
*O. viverrini*-IgG antibody							0.02			0.01
OD ≤ 0.24	32	18.8	109	31.0	1.0			1.0		
OD > 0.24	138	81.2	243	69.0	2.0	1.3–3.1		2.1	1.2–3.9	
*IL-1B* C-511 T polymorphisms							0.05			0.22
CC	40	23.6	69	19.5	1.0			1.0		
CT	99	58.2	187	52.8	0.9	0.6–1.4		0.8	0.4–1.5	
TT	31	18.2	98	27.7	0.5	0.3–0.9		0.4	0.2–0.8	
*TNF-A* G-308A polymorphisms							0.19			0.30
GG	105	61.8	244	68.8	1.0			1.0		
GA	29	17.1	58	16.3	1.1	0.7–1.8		1.2	0.6–2.4	
AA	36	21.1	53	14.9	1.6	1.0–2.6		1.7	0.9–3.3	

^a^Crude odds ratios from matched case-control analysis

^b^Adjusted for smoking (no/yes), alcohol drinking (< 14/≥14 units of alcohol per month), dietary consumption: dish of raw freshwater fish, processed beef, papaya salad (non-consumer, < 1/month & monthly, weekly, daily), total fruits (< 52/≥52 average times per month), and total vegetables (< 35/≥35 average times per month)

**Table 3:** Interactions of *O. viverrini* infection intensity with *IL-1β* and *TNF-α* polymorphisms on the risk of intrahepatic cholangiocarcinoma in Thailand

Polymorphisms	*O. viverrini* infection intensity	Cases	Controls	OR^a^	95% CI	*P*-value	*P*-value^b^
		*n*	*n*				
*IL-1B* C-511 T	IgG antibody						0.52
CC	OD ≤ 0.24	8	27	1.0			
CT	OD ≤ 0.24	17	56	0.9	0.4–2.5	0.90	
TT	OD ≤ 0.24	7	26	0.9	0.3–3.1	0.88	
CC	OD > 0.24	32	42	2.5	0.9–6.6	0.06	
CT	OD > 0.24	82	129	2.1	0.9–4.9	0.10	
TT	OD > 0.24	24	72	1.1	0.4–2.8	0.89	
*TNF-A* G-308A	IgG antibody						0.64
GG	OD ≤ 0.24	18	73	1.0			
GA	OD ≤ 0.24	5	20	0.9	0.3–2.8	0.87	
AA	OD ≤ 0.24	8	14	2.2	0.8–6.0	0.14	
GG	OD > 0.24	87	169	2.1	1.2–3.7	0.01	
GA	OD > 0.24	24	38	2.4	1.1–4.9	0.02	
AA	OD > 0.24	27	37	2.8	1.4–5.8	0.01	

^a^Crude odds ratios from matched case-control analysis

^b^*P* -value for interaction

## Discussion

In this work, our results successfully demonstrated the risk factors for ICC. For example, *O. viverrini* infection intensity detected by the IgG antibody was a risk factor for ICC and was comparable to previously reported studies in Northeast Thailand – Khon Kaen, [21] Nakhon Phanom [22] and Ubon Ratchathani [23]. Results obtained in this work showed the modified effects of *O. viverrini* infection with *TNF-α* codon 308 AA variant. Participants with only *O. viverrini* infection intensity had adjusted OR 2.1 (95% CI: 1.2–3.9), but participants who had both *O. viverrini* infection intensity together with AA variant of *TNF-α* had an increased OR of 2.8 (95% CI: 1.4–5.8).

The genotype frequencies of *IL-1β* C-511 T and *TNF-α* G308A polymorphisms found in the controls were consistent with other studies in Thailand [16, 24]. Prevalence of C and T alleles of *IL-1β* codon 511 were 45.9% vs. 46.4% and 54.1% vs. 53.6%, respectively. In addition, the allele distribution of *TNF-α* G308A was also consistent as reported before [24].

Very limited information exists in Thailand that reported the associations of *IL-1β* C-511 T and *TNF-α* G-308A polymorphisms with the risk of various cancers, especially in chronic hepatitis B virus infection-linked HCC [16, 24]. Although, there was no study about the association of *IL-1β* C-511 T and *TNF-α* G308A polymorphisms on ICC risk until now, the study on cytokines expression related cancer has been reported which acts as a diagnostic marker for cancer. The detection of serum levels of cytokines (such as IL-6 or IL-10) may be linked to the process of carcinogenesis or poor prognosis [25–28]. In our current study of ICC, the role of TNF-α in inducing epithelial-mesenchymal transition (EMT) of ICC cells has been published recently [6]. Moreover, the profile of cytokine production in peripheral blood mononuclear cells collected from subjects with and without *O. viverrini* infection was evaluated. Eleven cytokine profiles (IFN-γ, IL-1β, IL-2, IL-4, IL-5, IL-6, IL-8, IL-10, IL-12p70, TNF-α and LT-α), measured by flow cytometry, revealed that both pro-inflammatory and anti-inflammatory cytokines were increased in the *O. viverrini*-associated ICC compared to uninfected normal controls [29].

The basis of diagnosis of ICC was rarely done by histology (6.9%), and it is possible that other cases of fatal liver disease showing features of biliary obstruction, with a low serum alpha-fetoprotein, were possibly included in the case group. Despite this, the overall results support our hypothesis and are a pivotal factor in formulating experiments for future projects.

## Conclusions

There was a known association between the polymorphisms of *IL-1β* and *TNF-α*. Polymorphisms of *IL-1β* C-511 T and *TNF-α* G-308A are not a risk of ICC but an individual with *O. viverrini* infection has effects on all genotypes of the *TNF-α* gene (GG, GA, AA) that might promote ICC. Primary prevention of ICC in high-risk areas is based on efforts to reduce *O. viverrini* infection.

## Abbreviations

ASR: Age-standardized incidence rate
ELISA: Enzyme linked immunosorbent assay
HCC: Hepatocellular carcinoma
ICC: Intrahepatic cholangiocarcinoma
IL: Interleukin
KKCS: Khon Kaen Cohort Study
NHSO: National Health Security Office
*O. viverrini*: *Opisthorchis viverrini*
OD: Optical density
OR: Odds ratios
PCR-HRM: Polymerase chain reaction with high resolution melting analysis
TNF: Tumor necrosis factor
